# Native Infective Endocarditis: A State-of-the-Art-Review

**DOI:** 10.3390/microorganisms12071481

**Published:** 2024-07-19

**Authors:** Francesco Nappi

**Affiliations:** Department of Cardiac Surgery, Centre Cardiologique du Nord, 93200 Saint-Denis, France; francesconappi2@gmail.com or f.nappi@ccn.fr; Tel.: +33-149334104; Fax: +33-149334119

**Keywords:** infective endocarditis, native valve endocarditis, vegetation, gram-positive bacteria

## Abstract

Native valve infective endocarditis (NVE) is a global phenomenon, defined by infection of a native heart valve and involving the endocardial surface. The causes and epidemiology of the disease have evolved in recent decades, with a doubling of the average patient age. A higher incidence was observed in patients with implanted cardiac devices that can result in right-sided infection of the tricuspid valve. The microbiology of the disease has also changed. Previously, staphylococci, which are most often associated with health-care contact and invasive procedures, were the most common cause of the disease. This has now been superseded by streptococci. While innovative diagnostic and therapeutic strategies have emerged, mortality rates have not improved and remain at 30%, which is higher than that for many cancer diagnoses. The lack of randomized trials and logistical constraints impede clinical management, and long-standing controversies such as the use of antibiotic prophylaxis persist. This state of the art review addresses clinical practice, controversies, and strategies to combat this potentially devastating disease. A multidisciplinary team will be established to provide care for patients with presumptive NVE. The composition of the team will include specialists in cardiology, cardiovascular surgery, and infectious disease. The prompt administration of combination antimicrobial therapy is essential for effective NVE treatment. Additionally, a meticulous evaluation of each patient is necessary in order to identify any indications for immediate valve surgery. With the intention of promoting a more comprehensive understanding of the procedural management of native infective endocarditis and to furnish clinicians with a reference, the current evidence for the utilization of distinct strategies for the diagnosis and treatment of NVE are presented.

## 1. Introduction: An Overview of the Epidemiological, Pathophysiological, and Clinical Features of Disease

Native Valve Endocarditis is rare, with a frequency of approximately 2 to 10 cases per 100,000 person-years, making it a significant concern in clinical practice [1,2,3,4]. It is thought to be caused by injury to the endothelial or endocardial lining of the valve. In most cases, the presumed initial event is the lesion of the valvular endothelium or endocardium. This lesion results in the exposure of subendothelial collagen, as well as other matrix molecules, which then serve as a substrate for the formation of a complex conglomerate composed of platelets and fibrins. This process results in the formation of a microthrombotic lesion, which is medically referred to as a sterile vegetation. This can lead to a number of complications. Consequently, bacteria that are present in the circulatory system adhere to and colonize the aforementioned injured structure. A lack of an efficacious response by the host organism to the infection results in the replication of the bacterium within the lesion, which in turn stimulates further platelet and fibrin deposition. This process culminates in the formation of an infected vegetative structure, which is a defining feature of infective endocarditis [4,5].

IE is a disease that has been known for centuries, but it has evolved over time. In the past, it typically affected young or middle-aged adults with underlying rheumatic heart disease or congenital heart disease (CHD) [1,2,3].

With the advent of antibiotics, the decline of rheumatic heart disease, and the remarkable advances made in medicine throughout the twentieth century, a significant change occurred in the risk profile of IE. This shift was accompanied by notable shifts in patient demographics and microbiology. Prosthetic valve replacement, hemodialysis, venous catheters, immunosuppression, and intravenous drug use (IVDU) emerged as the principal risk factors [1,6]. The average patient was older and frailer, with an increasing number of comorbidities. In parallel, staphylococci surpassed oral streptococci as the most prevalent causative microorganism [1,6].

The 21st century has witnessed the continued evolution of IE, with the condition now affecting >25% of cases [1]. Concurrently, advances in cardiology have driven further changes in patient demographics and the manifestations of the disease. Alongside the emergence of cardiac implantable electronic devices (CIEDs), the incidence of infective endocarditis (IE) affecting complex devices has significantly increased [1,2].

**Figure 1:**
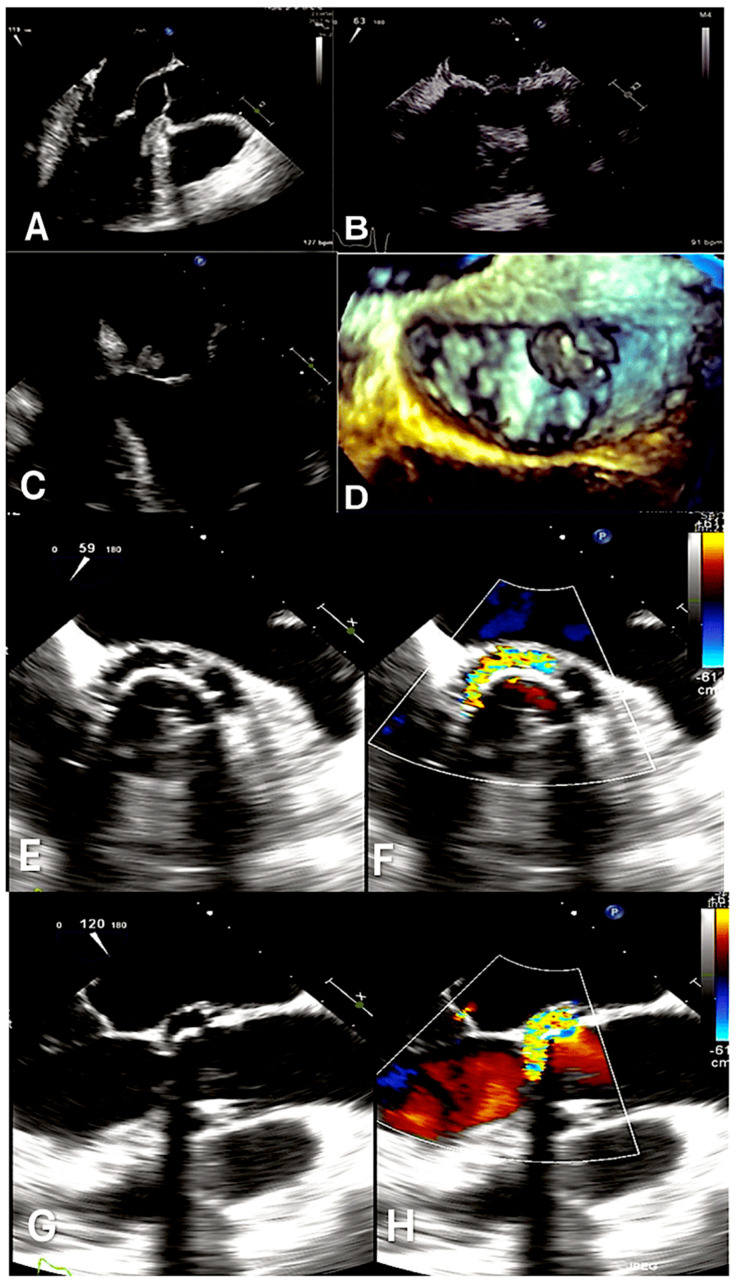
The following section reviews the echocardiographic findings in mitral valve endocarditis. (**A**) depicts a representative example of the normal mitral valve in two-dimensional transesophageal echocardiography, with the probe rotated to 120°. The thinner leaflets of the mitral valve are visible in the open position, in mid-diastole, with the aortic valve in the closed position. The left atrium is of an appropriate size, without any enlargement, and the left ventricular wall thickness is also within normal limits. (**B**) Mitral xenograft endocarditis. (**C**) Two-dimensional transesophageal echocardiography displays a vegetation on the atrial aspect of the anterior leaflet. (**D**) Three-dimensional reconstruction of the mitral valve (surgeon’s view) depicts the vegetation in C. Prosthetic valve endocarditis is evident. (**E**,**F**) The images presented are those obtained from the short-axis view (59 degrees of probe angle) and (**G**,**H**) the long-axis view (120 degrees). The images are presented in two different ways: with (**A**) and without color-Doppler analysis. The color doppler analysis was performed on a mechanical aortic prosthesis with a posterior semilunar abscess, which involved the aortomitral junction.

The prevalence of mitral valve involvement is lower than that of the aortic valve, according to a review of observational data on IE. In a report that examined 775 patients, it was found that 51% of cases exhibited involvement of the aortic valve. Of these, 59% were classified as prosthetic valve endocarditis (PVE) and 68% as invasive endocarditis [4]. In contrast, mitral valve involvement was observed in 30.7% of cases, with PVE representing 29% and invasive endocarditis 35%. In an additional study, the prevalence of aortic valve involvement was found to be 47%, while mitral valvular involvement was identified to be 31% [4]. Figure 1 depicts a xenograft mitral valve endocarditis.

The French registry indicates a prevalence of IE that ranges from 43.8% to 35.4%. Active infective endocarditis may present in two forms: NVE or prosthetic valve endocarditis (PVE). NVE has been estimated to affect around 50% of cases, while PVE affects approximately 10%. The infection can affect a single valve, multiple valves in various combinations, or the entire heart structure. The extent of infection and the resulting damage depend on several factors, including the location of the affected valves and the degree of extension into the heart structures. In certain instances, the infection may result in extensive destructive damage to other valve components. In instances where the aortomitral curtain has been extended or there is a co-occurrence of two or more valves, which occurs in approximately 40% of cases, the mortality rates before and after the procedure increase significantly [4,5].

The vegetative microenvironment is weakly affordable to neutrophils and host defense molecules due to the protective effect it exerts. Vegetations are replete with bacteria at exceedingly high densities, with colony forming units (CFU) numbers in the range of 109 to 1010 per gram of vegetation. These bacteria have been found to facilitate high-grade bacteremia and the subsequent proliferation of the vegetation, which undergoes a transformation from a solid to a liquid state, becoming friable and readily fragmented into the circulation. Four mechanisms account for the majority of clinical manifestations of infective endocarditis. These include valvular destruction, paravalvular extension of infection and heart failure, microvascular and large vessel embolization, and metastatic infection of target organs. The affected organs include the brain, kidneys, spleen, and lungs. Additionally, immunologic phenomena such as hypocomplementemic glomerulonephritis and false positive serologic findings of rheumatoid factor, antineutrophil antibodies, or syphilis occur in response to the three conditions that drive the disease process: (1) high bacterial densities, (2) the growing vegetation, and (3) the friability and fragmentation of the vegetative growth [1,2,3,4,5].

Individuals with heart abnormalities are at an increased risk of developing infectious endocarditis. These abnormalities include congenital malformations such as ventricular septal defect and bicuspid aortic valve, as well as acquired valvular disease, such as degenerative valvular disease, aortic stenosis, and rheumatic heart disease. In contrast to low-income countries where rheumatic heart disease represents the most common predisposing feature for IE, it is unconventional in high-income countries where the most frequent predisposing cardiac conditions are degenerative valvular diseases, congenital valvular abnormalities, and intracardiac devices [3,4,5]. It is of paramount importance to consider non-cardiac risk factors when evaluating patients, which include intravenous drug users, hemodialysis treatment, bad dental hygiene, chronic liver disease, diabetes mellitus, compromised immunity, neoplastic disease, and the presence of indwelling intravascular devices.

Approximately 90% of patients with infective endocarditis present with a fever and a heart murmur, while 75% exhibit both of these symptoms [1,2,6,7]. Acute infective endocarditis in situ is known to manifest with a rapidly progressive course, which may, on occasions, be complicated by congestive heart failure, stroke, systemic or pulmonary embolization, severe sepsis, or septic shock. Alternatively, it may manifest subacutely with nonspecific symptoms such as low-grade fever, malaise, chills, sweats, dyspnea, back pain, arthralgias, and weight loss over a period of weeks or sometimes months. Furthermore, it should be acknowledged that microembolic or immunologic events such as splinter hemorrhages, conjunctival hemorrhages, Osler nodes, Janeway lesions, and Roth spots may be present in approximately 5 to 10% of cases [5].

### Key Points for Native-Valve Infective Endocarditis

- Modifications of the Duke criteria, based on findings from physical examination, echocardiography, microbiologic evaluations, and computed tomographic and magnetic resonance imaging of organ involvement, have been demonstrated to be both sensitive and specific for the clinical diagnosis of NVE.
- In the case where transesophageal echocardiography (TEE) is more sensitive than transthoracic echocardiography (TTE) for identifying valvular vegetations and periannular complications of NVE, TEE should be employed as the initial diagnostic tool. This is in contrast to TTE, which may yield false negative or nondiagnostic results.
- In the treatment of NVE caused by methicillin-susceptible *Staphylococcus aureus*, the use of beta-lactam antibiotics represents the first-line recommendation in preference to vancomycin or daptomycin.
- It can be postulated that in older individuals with NVE supported by *Enterococcus faecalis*, in particular those with underlying renal impairment or undergoing treatment with other nephrotoxic medication, ampicillin combined with ceftriaxone may prove to be a more appropriate choice than aminoglycoside containing schemes.
- The available evidence indicates that early surgical intervention for uncontrolled infection, congestive heart failure resulting from valvular failure, or the prevention of embolization in the central nervous system may be beneficial.
- It is reasonable to propose that in selected cases, a course of treatment involving the oral administration of drugs may be considered following an intravenous course of therapy.

## 2. Search Strategy and Selection Criteria

A comprehensive search of MEDLINE, Embase, and the Cochrane Library was conducted using the search terms “endocarditis” or “native infective endocarditis” in conjunction with “epidemiology”, “pathogenesis”, “manifestations”, “imaging”, “treatment”, “surgery”, or “device”. Publications from the past ten years were primarily selected, though older, widely referenced, and highly reputable works were not excluded.

Additionally, a comprehensive search of the references of all articles identified through our search strategy was conducted. Articles deemed to be relevant were selected and included in our review. For readers who may require more details or background information, we have provided citations for recommended review articles.

## 3. Microbiological Characteristics

**Figure 2:**
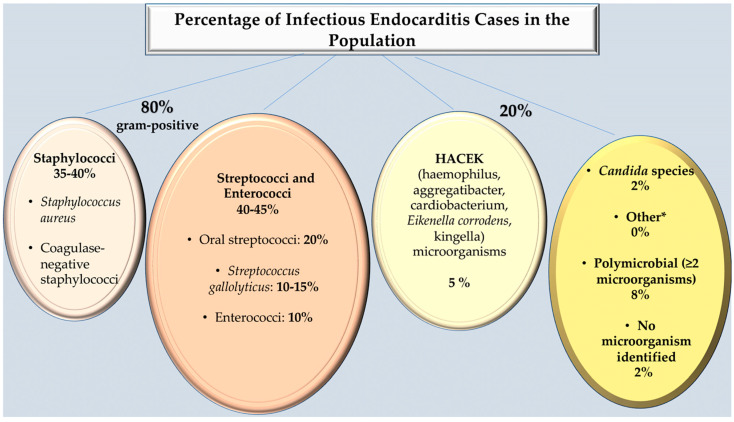
Percentage of Infectious Endocarditis Cases in the Population. * Fungal endocarditis, usually *Candida* or *Aspergillus*, is rare but often fatal, arising in patients who are immunosuppressed or after cardiac surgery, mostly on prosthetic valves. Includes small numbers of Enterobacteriaceae, *Propionibacterium acnes*, *Coxiella burnetii*, *Bartonella quintana*, *Tropheryma whipplei*, *Gordonia bronchialis*, *Bacillus* spp., *Erysipelothrix rhusiopathiae*, *Neisseria elongata*, *Moraxella catarrhalis*, *Veillonella* spp., *Listeria monocytogenes*, *Acinetobacter ursingii*, *Campylobacter fetus*, *Francisella tularensis*, and *Pseudoonas aeruginosa*, *Lactobacillus* spp., *Corynebacterium* spp., *Catabacter hongkongensi.* Refs. [1,2,3,4,5,6,7,8,9,10].

It is estimated that gram-positive bacteria account for approximately 80% of cases of NVE worldwide. In the majority of cases of native-valve infective endocarditis, bacteria are identified. These may include staphylococci (such as *Staphylococcus aureus*) in 35–40% of cases, streptococci (such as *Viridans streptococci*, which are present in approximately 20%, and *Streptococcus gallolyticus*, previously identified as *S. bovis*, which is found in approximately 15%, or enterococci (which are present in approximately 10%) [1,2,3,4]. It would be remiss of us not to mention that coagulase-negative staphylococci, a common cause of prosthetic-valve infective endocarditis, are uncommon in native-valve infective endocarditis, except for *S. lugdunensis*, which may be similar to *S. aureus* in terms of clinical presentation. It is important to note that in 5% of cases, HACEK species are isolated, including *Haemophilus* species, *Aggregatibacter* (formerly *Actinobacillus*) species, *Cardiobacterium* species, *Eikenella corrodens*, and *Kingella* species. In addition, fungi, polymicrobial infections, and, in very rare instances, aerobic gram-negative bacilli are also identified [1,2,3,8,9,10]. Figure 2 presents the percentage of infectious endocarditis cases in the population, which serves to illustrate the pressing need for the development of improved treatment strategies.

The requisite momentum for the production of biofilms and the impact of these on the maturation of antibiotic tolerance have been the subject of study. The results showed that benchmark strains of *Staphylococcus aureus*, as well as three clinical isolates of infective endocarditis, formed biofilms that mimicked IE vegetation six hours after the onset of infection. As a result, the sooner the antibiotic was initiated, the more pronounced was its pharmacological effect in limiting the maturation of the biofilm. This is an indication that timely treatment is more effective at stopping the disease from spreading. A microscopic observation of the biofilm formation process was conducted by studying the growth of bacterial aggregates on the vegetation of IE in vitro (test) modeling and by examining the interaction of the aggregates with an antibiotic. The formation of mature, antibiotic-resistant biofilms was observed six hours after initial inoculation, prompting the search for optimal treatment strategies for IE [5,7].

In consideration of the mounting prevalence of antibiotic resistance, there has been a discernible surge in the focus of research efforts on microbiological analysis, with a particular emphasis on the exploration of bacterial factors as potential therapeutic targets for immunomodulation. This conclusion is derived from the recognition of the pivotal role of bacterial factors in an organism’s ability to colonize, infect, and ultimately cause disease [5,7,9].

Proteins in the adhesive matrix molecules (MSCRAMM) have recently received significant attention due to their widespread presence and unique ability to promote the initiation of infections, including endocarditis, in both traditional and opportunistic pathogens. Their central role in these processes is of particular interest. It is unfortunate that complications have been encountered in the process of isolating and characterizing MSCRAMM from *E. faecalis*, which has yielded minimal success. This is likely due to the lack of adherence to ECM proteins observed in this microorganism under the laboratory growth conditions. In contrast, other relatives of *E. faecalis*, including staphylococci and streptococci, have been observed to display greater invasiveness [5,7,9].

To tackle the aforementioned challenge, a computational approach based on bioinformatics was employed in order to identify a number of proteins that are predicted to form MSCRAMM-like structures. The researchers proceeded to evaluate their reactivity with sera derived from patients infected with *E. faecalis*. This evaluation led them to conclude that some of the predicted proteins were indeed expressed by *E. faecalis* during infection. In particular, the investigation sought to examine antibodies present in the sera of patients diagnosed with endocarditis due to *E. faecalis*. Consequently, the demonstration of antibodies directed against three of these proteins, accompanied by markedly high antibody titers in the majority of infected patients’ sera, provides a foundation upon which further research may be conducted [5,7,9].

### Impact of Culture Negative

Infective endocarditis is a complex disease with a high rate of morbidity and mortality during hospitalization, particularly when uncontrolled or complex infection drives the patient towards the need for surgery. The management of IE is best approached through a multidisciplinary approach, including the formation of a dedicated endocarditis team, and by ensuring prompt access to advanced imaging techniques. Patients diagnosed with IE should undergo a comprehensive evaluation of their presenting symptoms and a transthoracic echocardiogram. The objective of this evaluation is to ascertain the presence and development of vegetations affecting one or more leaflets, as well as the extent of the infection in heart and aorta components. This includes the leaflet, annulus, trigones, intervalvular fibrous tissue, left atrium, and aortic root, along with the size and function of the left ventricle [1].

Approximately 10% of patients with IE demonstrate a lack of growth in blood cultures. This can lead to a missed diagnosis and an approximate 5.2% incidence of cases where a blood culture result is negative, as observed in a French population-based cohort of 497 patients. Three potential explanations can be postulated for this phenomenon. At present, the individual is receiving treatment with an antibiotic that may have an inhibitory effect on the growth of the microbe. Alternatively, it is possible that the infection is being supported by other pathogens belonging to the *Bartonella* species, *Brucella* species, *Coxiella burnetii*, and a group of bacteria known as the HACEK family (*Haemophilus* species, *Actinobacillus actinomycetemcomitans*, *Cardiobacterium hominis*, *Eikenella corrodens*, and *Kingella kingae*). The time for detecting infection is of the utmost importance. If cultures are negative at 5 days, serological testing for Coxiella and Bartonella should be conducted, and if they are also negative, testing for Brucella, Mycoplasma, Legionella, and Chlamydia should be undertaken. Extended blood culture after 7 days provides no further useful yield, even for the HACEK bacteria, which are characteristically slow growing. Another factor to consider is non-bacterial thrombotic endocarditis, which is a complication of advanced cancer [1,2,4,5].

Microorganisms can be identified using serological techniques such as valve polymerase chain reaction (PCR) assays or specialized microbiological techniques. These methods can successfully identify the pathogen in approximately two-thirds of cases. Specifically, the use of excised valve material in molecular techniques can serve as a valuable complement to other methods when available. One such technique is broad-range PCR, which is highly sensitive and can amplify minute quantities of conserved bacterial or fungal DNA. When combined with sequencing, it can facilitate the identification of the specific organism in question. In particular, this approach is of value in patients who have previously received antibiotic treatment since bacterial DNA is frequently persistent, and also in cases of non-cultivable pathogens including *T. whipplei*. However, PCR carries the inherent risk of a false-positive result due to the potential contamination of samples. Furthermore, it is possible for PCR to remain positive following the eradication of viable bacteria. Therefore, it should not be used as a basis for determining the prolonged in vivo efficacy of a given therapy. The integration of PCR with mass spectrometry, a technique that has recently emerged, has the potential to facilitate the direct characterization of bacteria present within blood and valve tissue samples [1,2,4,5,6,8].

It has been demonstrated that common serologic tests may lack the necessary sensitivity and specificity to accurately diagnose some strains of *Staphylococcus aureus* and *Streptococcus viridans*, both of which are known to exhibit extreme aggressiveness. Furthermore, this bacterium is challenging to detect even with advanced methodologies, such as MPB genotyping and matrix-assisted laser desorption/ionization time-of-flight (MALDI-TOF) systems. The persistence of negative blood culture results may delay the commencement of treatment, which can have a significant impact on a patient’s prognosis. In such cases, emergency surgery should be considered if clinical conditions are deteriorating [2,4].

The extended period of time required by internists to reach a diagnosis frequently stems from the challenge of identifying the pathogen responsible. In instances where aggressive and non-detectable microorganisms are present, the administration of nonspecific antibiotic therapies over 24 to 48 h may result in the extension of lesions involving multiple valves, as well as the destruction of a considerable proportion of the cardiac area, with the potential for adverse outcomes. A paradigmatic exemplum of this phenomenon is represented by intracellular microorganisms that infect humans, such as *Coxiella burnetii, Bartonella* species, or *Tropheryma whipplei*. In such instances, both the degree of in vivo antigen display and the in vivo immune response status of the host become crucial factors [2,4].

**Figure 3:**
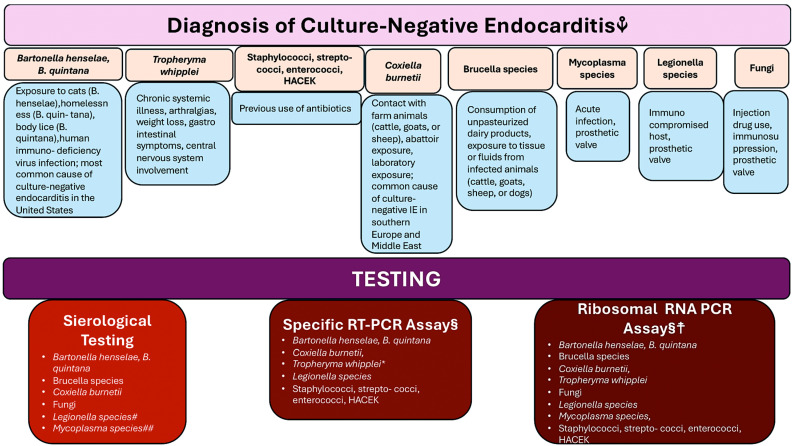
The diagnosis of culture-negative endocarditis and related testing to detect microorganisms in the red boxes are presented. The pink box illustrates the microorganisms and related clinical and epidemiologic clues (Box blue). The varying coloration of the red box signifies the type of testing. ⚘ In the event that a dash is present, it signifies that the test to detect the microorganism in question is not available or not applicable at this moment in time. HACEK stands for *Haemophilus* species, *Aggregatibacter* (formerly *Actinobacillus*) species, *Cardiobacterium* species, *Eikenella corrodens*, and *Kingella* species. It also encompasses PCR polymerase chain reaction and RT-PCR reverse-transcriptase PCR; §, the sensitivity of the method is significantly greater if the RT-PCR or broad-range 16S or 18S RNA PCR assay is conducted on valvular vegetation or abscess material, in comparison with the use of blood as a specimen; ☨ PCR assays that cover a broad range of targets often include the 16S and 18S ribosomal RNA genes; # The tests for Legionella pneumophila serotype 1, as indicated by sierologic tests and urinary antigen tests, are the only tests capable of detecting the aforementioned serotype; ## Serologic tests are employed solely for the purpose of detecting the presence of Mycoplasma pneumoniae; * In the event of an extracardial lesion, a biopsy of the affected tissue (e.g., small bowel and synovium, if present) is recommended. Refs. [2,4,6,8].

In the context of uncontrolled or complex infection, which represents the second indication for surgery, culture-negative results may contribute to a more complex picture. The potential for infection to spread beyond the valve annulus may result in the formation of an abscess, pseudoaneurysm, fistula, or atrioventricular block. A pseudoaneurysm is defined as a perivalvular cavity that communicates with the cardiovascular lumen, which can be identified by color flow Doppler echocardiography. In contrast, an abscess is a thickened, pus-filled cavity that lacks such communication [1,4,9,10]. Progressive perivalvular infection may lead to fistula formation. Ultrasonic evidence of aorto-cavitary formation is associated with a mortality rate exceeding 40%, even in cases where surgical intervention is employed. Persistent or relapsing infection, or infection caused by aggressive or antibiotic-resistant microorganisms, including *S. aureus*, *S. lugdunensis*, pseudomonas, and fungi, also warrant surgical intervention [2,4]. In Figure 3, the diagnosis of culture-negative endocarditis and the subsequent tests employed to identify microorganisms are presented.

## 4. Strategy Assessment and Proof of Concept

**Figure 4:**
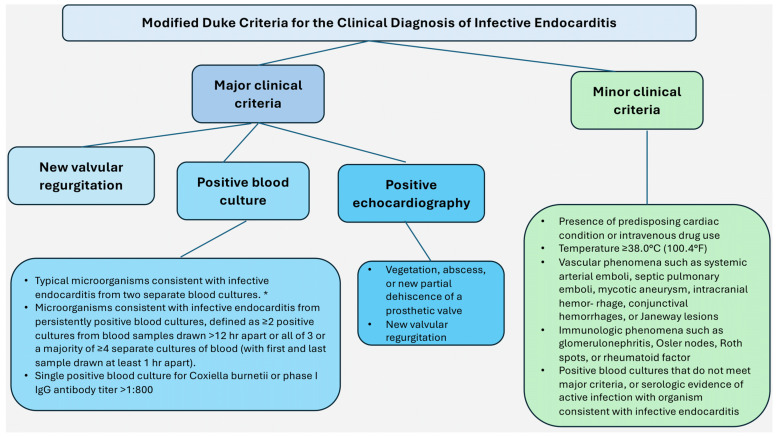
Modified Duke Criteria. * Staphylococcus aureus, Viridans streptococci, Streptococcus gallolyticus, HACEK (Haemophilus species, Aggregatibacter (formerly Actinobacillus) species, Cardiobacterium species, Eikenella corrodens, and Kingella species), and community-acquired enterococci in the absence of a primary focus. Refs. [14,15,28].

The modified Duke criteria serve as the fundamental basis for the diagnosis of IE. If the infective pathogens are identified by histological analysis or culture of the vegetations, intracardiac abscesses, or peripheral emboluses, a final pathological diagnosis can be made. Alternatively, a confirmed pathological diagnosis can be made if the histologic evidence of vegetation or intracardiac abscess is validated by an analysis that demonstrates active endocarditis [11,12,13]. The diagnosis of infective endocarditis is made on the basis of a combination of both objective and subjective criteria, which are derived from microbiologic, echocardiographic, and clinical data with regard to sensitivity, it is possible to estimate that the modified Duke criteria for infective endocarditis are approximately 80% effective for cases that are definitively diagnosed and higher if possible cases are included. This is based on data from landmark studies by Habib et al. [14] and Li et al. [15]. In their observations, Habib et al. [14] determined that 24% of patients who had been definitively diagnosed with IE continued to be incorrectly classified as “possible IE”, particularly when culture-negative and Q fever were suspected. They recommended that the diagnostic value of echographic criteria be increased in patients who had received prior antibiotic treatment, exhibited typical echocardiographic findings, and considered the serologic diagnosis of Q fever as an important criterion for IE diagnosis. This could further enhance the clinical diagnosis of IE. A study conducted by Li et al. [15] underscores the importance of validating diagnostic criteria, such as those proposed by Duke. Although the sensitivity and specificity of Duke’s criteria for diagnosing IE have been validated by investigators in Europe and North America, several limitations remain. The Duke IE database, which contains >800 cases of both confirmed and probable IE from 1984 onward, provides valuable insights into the effectiveness of this approach. In addition, databases on echocardiograms and patients with *Staphylococcus aureus* bacteremia at Duke University Medical Center are also maintained. This experience with the Duke criteria in clinical practice and analyses of these databases and of the work of others have led to the proposal that the following modifications be made to the Duke schema: the category “possible IE” should be redefined as having at least one major criterion and one minor criterion or three minor criteria. In light of the widespread use of transesophageal echocardiography (TEE) and the increasing prevalence of *S. aureus* bacteremia, it seems prudent to eliminate the minor criterion “echocardiogram consistent with IE but not meeting major criterion”. Furthermore, given that bacteremia due to *S. aureus* is now recognized as a major criterion in the absence of either a nosocomially acquired infection or a removable source of infection, it would be advantageous to consider this as such. Similarly, positive Q-fever serology should be elevated to the status of a major criterion, given that it is a well-established risk factor for IE [15]. The modified Duke criteria are illustrated in Figure 4.

It should be noted that the aforementioned criteria exhibit diminished sensitivity when employed in the context of infections associated with prosthetic valves or cardiac devices. These infections are particularly challenging to diagnose and include those developing in the right side of the heart and those presenting with culture-negative infective endocarditis [10,14,16,17,18]. In the case of non-compliance with the criteria for either definite or probable infective endocarditis, the negative predictive value is approximately 90 percent.

Blood cultures represent a crucial diagnostic tool for the identification and treatment of infective endocarditis. Moreover, they fulfill a primary criterion established by the Duke criteria. The choice and dosing of antimicrobial agents largely depend on the blood culture isolate and its antimicrobial susceptibility profile. In approximately 90 to 95% of cases of NVE, a positive blood culture result is observed. To optimize recovery of a pathogen, a strategy involving the acquisition of three distinct sets of blood cultures at 30-min intervals before the administration of antibiotics has proven to be effective [1,19,20]. The administration of antimicrobial therapies represents the most common cause of a negative blood culture result. Other potential etiological agents that can lead to a negative blood culture result include those pathogens that are unable to grow effectively or at all within the conventional parameters of standard blood culture media. These include *Bartonella* species, *Coxiella burnetii*, *Tropheryma whipplei*, and *Legionella* [1,4,21]. Another factor that can result in a negative blood culture result is suboptimal specimen collection [22].

Should a blood culture yield negative results, it is advisable to proceed with serologic and molecular testing for pathogens that are likely to be responsible. This testing is guided by epidemiological clues, such as the association between *C. burnetii* infection and exposure to farm animals, and between *B. quintana* infection and homelessness.

In adults, *Bartonella* spp. has emerged as the most common cause of blood culture-negative IE. The clinical manifestation, as well as the pathological findings observed upon renal biopsy in patients diagnosed with Bartonella infection-associated glomerulonephritis, exhibit subtle distinctions and distinctive characteristics, when compared to other bacterial pathogens associated with glomerulonephritis. The two most commonly implicated species causing IE in humans are *Bartonella henselae* and *Bartonella quintana* [23,24]. A subacute presentation, which primarily affects damaged native and/or prosthetic heart valves, is often accompanied by high titers of anti-neutrophil cytoplasmic antibodies, with the majority targeting proteinase-3 specificity. The bacteria are also fastidious and lack positive blood cultures. In addition, there is a higher frequency of focal glomerular crescents compared to other bacterial infection-related glomerulonephritis, a distinctive feature of Bartonella IE-associated glomerulonephritis. C3-dominant immunofluorescence staining is frequently observed, although C1q and IgM staining is also present [23,24].

The study conducted by Kitamura et al. [25] revealed that a full-house immunofluorescence staining pattern has been observed in other cases, including those of infectious granulomatous nephritis caused by bacteria other than those associated with IE. The clinical presentation is characterized by non-specific generalized symptoms, cytopenia, heart failure, and organ damage due to embolic phenomena. These features require a multidisciplinary approach to management. It is crucial to be aware of the recently updated modified Duke criteria for IE, to have a high index of suspicion for underlying infection despite negative microbiologic cultures, to consider a history of exposure to animals, particularly cats, infected with *Bartonella* spp., and to utilize send-out serologic tests for *Bartonella* spp. early in the course of management in order to facilitate early diagnosis and initiate appropriate treatment [21,23,24,25].

Molecular diagnostics employs the PCR technology to amplify nucleic acids. This can be achieved through the utilization of particular primers targeting a particular species or genus, or broad-range primers that target the 16S ribosomal RNA (rRNA) gene for the detection of bacterial pathogens and the 18S rRNA gene for the identification of fungal pathogens.

With regard to PCR-based diagnostic tests, sensitivities have been reported to range from approximately 33 to 90%, while specificities are estimated to lie between approximately 77 and 100%. These in vitro results have been documented by Fournier et al. [21] and Liesman et al. [26] Next-generation sequencing technology, which is expected to be more accurate than polymerase chain reaction-based approaches, is anticipated to gain prominence in the coming years. For molecular assays, the preferred specimen is an excised valve or vegetable. In cases where the pathogen is difficult to determine, plasma DNA amplification assays may facilitate microbial diagnosis.

Echocardiography is a fundamental diagnostic and management vehicle in the evaluation and treatment of infective endocarditis [27,28,29,30,31,32,33]. Transthoracic echocardiography (TTE) exhibits a sensitivity for detecting vegetations in native-valve infective endocarditis ranging between 50 and 60%. However, TEE has an enhanced sensitivity, with a reported range of 90% or higher [13,14,15]. Approximately 95% of the characteristics of the two are identical. Given that TTE has lower sensitivity in identifying intracardiac complications (e.g., paravalvular abscess), TEE is the preferred imaging modality to exclude infectious process in individuals suspected to have this condition and to assess for intracardiac complicating advents [10,27,28].

**Figure 5:**
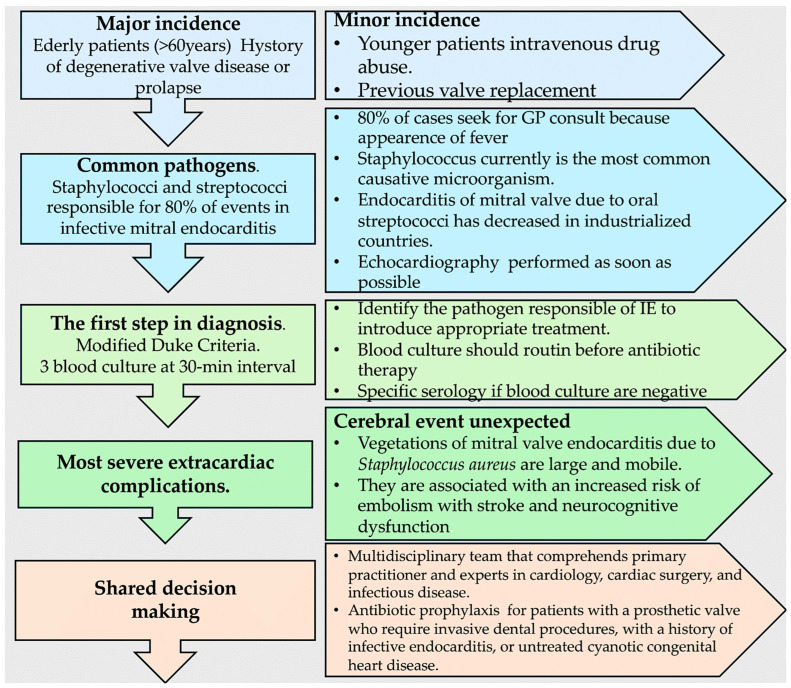
Clinical evaluation and diagnosis flowchart. Refs. [4,5,6,7,8,9,10,11,12,13,14,15,16,18,19,20,21,22,23,24,25,26,27,28,29,30,31,32,33,34].

**Figure 6:**
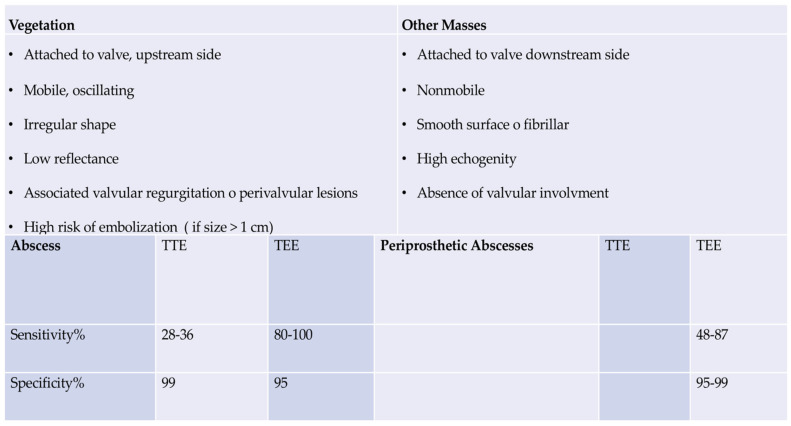
Sensitivity and specificities of echocardiography in the detection of abscesses. Refs. [27,28,31,32]. From Nappi et al. Bridging Molecular and Clinical Sciences to Achieve the Best Treatment of *Enterococcus faecalis* Endocarditis. *Microorganisms* **2023**, *11*, 2604. Ref. [42].

Among the more recent developments in imaging technology, the 18F-fluorodeoxyglucose cardiac positron-emission tomography (PET) and computed tomography (CT) combination represents one of the most extensively researched techniques [34,35]. PET-CT is the most suitable method for the diagnosis and evaluation of prosthetic-valve infective endocarditis. It is noteworthy that the role of this imaging investigation in native-valve infective endocarditis has not yet been sufficiently evaluated, and its efficacy is yet to be established [27,28,34,35,36]. Figure 5 presents a clinical evaluation and diagnosis flowchart, while Figure 6, which is presented below, outlines the efficacy of specific diagnostic imaging techniques for the detection of IE.

- *Clinical Evidence: Imaging Criteria*

Despite the existence of alternative imaging techniques, echocardiography continues to be the headline imaging modality for the identification of anatomical evidence of infective endocarditis, a condition for which the diagnostic criteria have been well-established [19,20,27]. Furthermore, it is a pivotal criterion in the 2023 Duke-ISCVID IE Criteria [27,32,33,37]. The presence of valvular vegetation represents the most prevalent echocardiographic manifestation of infective endocarditis. However, more complicating events affecting the leaflets of valves (e.g., perforation and pseudoaneurysm), paravalvular structures (e.g., abscess and fistula), or prosthetic valves (e.g., valvular dehiscence) have been identified as potential indicators of IE [27,28]. It has been demonstrated that TEE is a less sensitive diagnostic modality for IE than TEE. Consequently, TEE is de facto required for the diagnosis of IE, particularly in cases involving prosthetic valves, cardiac devices, or probable complications such as perforation, para-valvular lesions, fistula, prosthetic-valve dehiscence, and hematogenous spondylodiscitis. These indications are in alignment with recommendations outlined in literature [27,28,29,30,32,33,37].

TEE is a diagnostic tool that assesses the development and progression of abscesses, as well as the mechanism and severity of valve regurgitation. It is notable that TTE demonstrates moderate sensitivity (75%) and specificity (>90%) in the detection of vegetation, which is indicative of the probable occurrence of NVE. This finding is consistent with the results of previous studies, including those by [28,30,38].

In the event that a patient does not present evidence of infection on TTE, either in negative or inconclusive form, and yet exhibits a high likelihood of having IE, it is recommended that they undergo TEE, given that it has a sensitivity in excess of 90%.

A negative TEE indicating the absence of vegetations is a reliable indicator of the absence of disease. However, in the event of a high level of clinical suspicion, a repeat examination seven to ten days later is required to confirm the initial negative result. In the event that the test remains negative, it becomes possible to exclude the diagnosis of IE. The administration of an additional echocardiography would not result in the acquisition of any further beneficial data. As the test’s specificity is not 100% accurate, it is essential to exclude false positives for a more nuanced differential diagnosis [10,18,27,28,30,31,32,38,39,40,41]. Figure 6 [27,28,31,32].

The current recommendation, based on the available evidence, is that the diagnostic process should include the use of a CT scan, an 18F-FDG-PET/CT, and a cardiac MRI. In 2023, the ISCVI Working Group included cardiac computed tomography (CCT) as a supplementary imaging modality in its Duke-ISCVI IE Criteria. Although cardiac computed tomography (CCT) has inferior capability for the detection of vegetations compared to echocardiography, it demonstrates enhanced sensitivity in the detection of paravalvular lesions, due to its superior spatial resolution [27,39,40,42,43].

**Figure 7:**
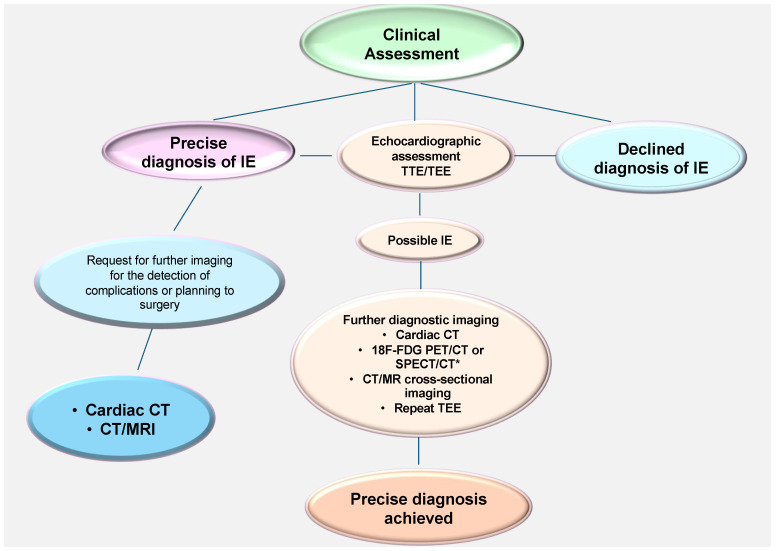
Clinical assessment of IE. To diagnose IE, a series of imaging techniques may be employed, including TTE, TEE, CT, and CT/MRI. These techniques are employed in a stepwise manner to either confirm or exclude the presence of an infection. The use of 18-FDG PET-CT or SPECT/CT has been demonstrated to have a high degree of specificity for the identification of NVEs. Abbreviations; CT, computed tomography; 18F- FDG PET/CT, positron emission CT with 18F-fluorodeoxyglucose; IE, infective endocarditis; MRI; magnetic resonance imaging; NVE; native endocarditis; TEE; transesophageal echocardiography; TTE, transthoracic echocardiography. * 2023 Duke-ISCVID IE Criteria. Ref. [28].

The 2023 Duke-ISCVID IE Criteria now include positron emission CT with 18F-fluorodeoxyglucose ([18F] FDG PET/CT) as an imaging modality. [18F] FDG PET/CT has been demonstrated to outperform echocardiography in the evaluation of prosthetic material, leading to the reclassification of a significant proportion of suspected perivalvular leakage cases from the “possible” to the “definite” category of IE. Given the continued debate surrounding the efficacy of [18F] FDG PET/CT in ruling out infective endocarditis (IE), the International Society of Cardiovascular Disease Investigators (ISCVID) Working Group has prioritized research into the test’s positive predictive value. The incorporation of [18F] FDG PET/CT as a primary criterion within the Duke Criteria has been demonstrated to markedly enhance the identification of definitive PVE (pooled sensitivity, 0.86 (0.81–0.89]; pooled specificity, 0.84 (0.79–0.88)) in comparison to echocardiography alone [27,28,44]. In the context of cardiac infections, [18F] FDG PET/CT holds particular significance for the diagnosis of such conditions in patients with intricate cardiac implants, including multiple prosthetic valves, combined aortic valves and grafts, and congenital heart disease [27,28,31,44,45,46]. In Figure 7, the clinical assessment of IE using imaging criteria is reported.

## 5. Clinical Use: Antimicrobial Therapies

**Figure 8:**
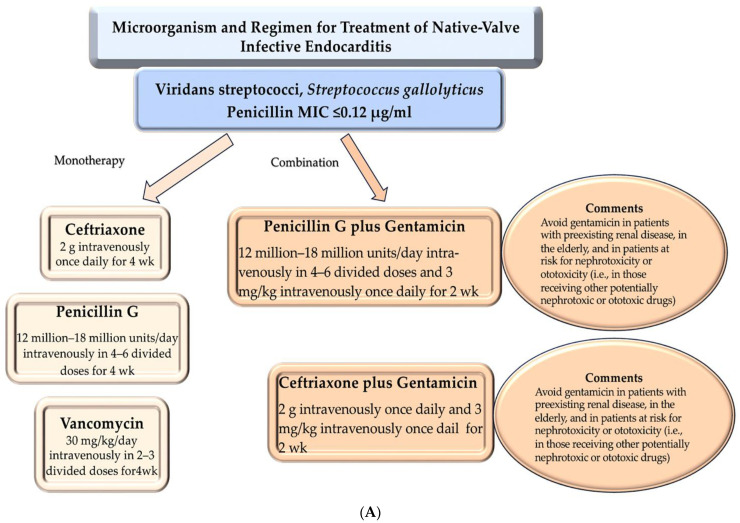

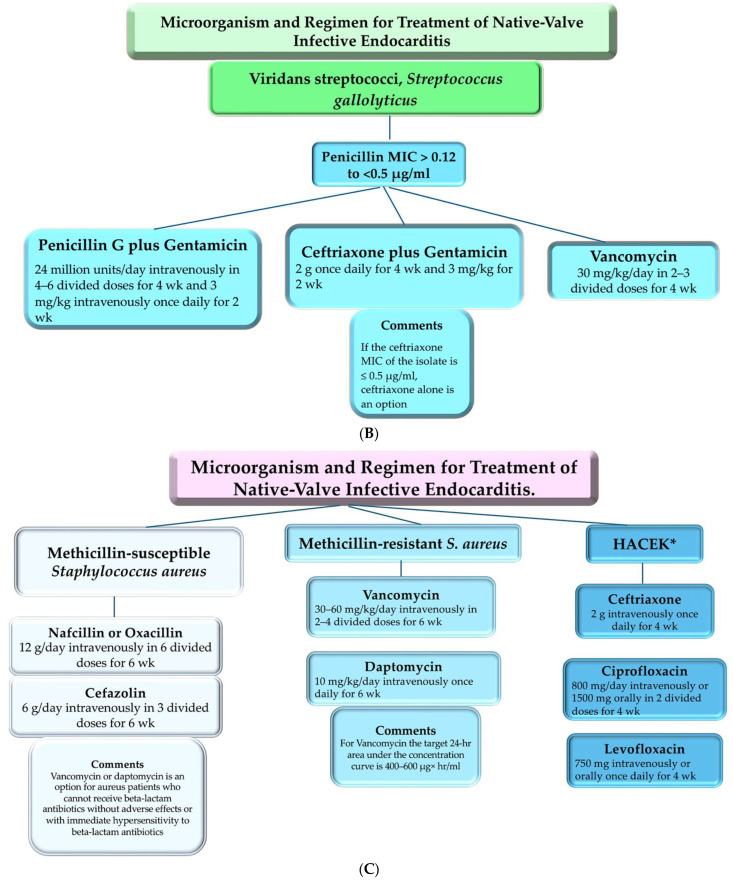

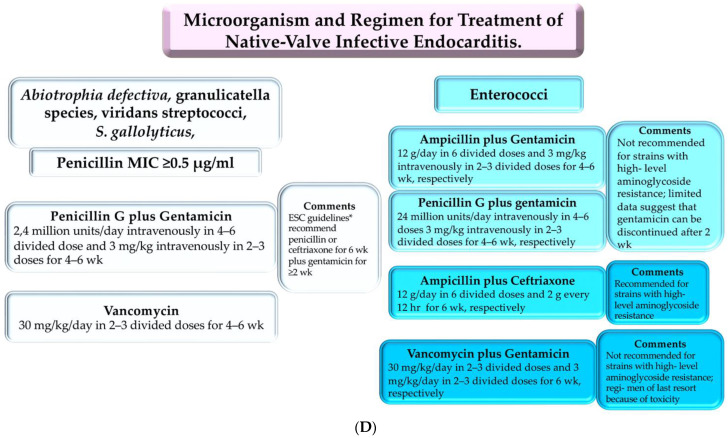
(**A**) This illustration demonstrates the efficacy of antibiotic treatment in NVEs for *Viridans streptococci* and *Streptococcus gallolyticus* at a penicillin MIC of ≤0.12 μg/mL. The duration of therapy once blood cultures have converted to negative is shown. Abbreviations; MIC, minimal inhibitory concentration; wk, week [27,46,47,48,49,50,51,52,53,54,55,56,57,58,59,60,61,62,63,64,65,66,67,68,69,70,71,72]. (**B**) This illustration demonstrates the efficacy of antibiotic treatment in NVEs for *Viridans streptococci* and *Streptococcus gallolyticus* at a penicillin MIC of >0.12 to <0.5 μg/mL and for enterococci. The duration of therapy once blood cultures have converted to negative is shown. Abbreviations; MIC, minimal inhibitory concentration, wk, week [27,46,47,48,49,50,51,52,53,54,55,56,57,58,59,60,61,62,63,64,65,66,67,68,69,70,71,72]. (**C**) This illustration demonstrates the efficacy of antibiotic treatment in NVEs for methicillin-susceptible *Staphylococcus aureus*, methicillin-resistant *S. aureus* and HACEK. The duration of therapy once blood cultures have converted to negative is shown. Abbreviations; MIC, hr, hour; wk, week. * HACEK denotes *Haemophilus* species, *Aggregatibacter* (formerly *Actinobacillus*) species, *Cardiobacterium* species, *Eikenella corrodens*, and *Kingella* species [27,46,47,48,49,50,51,52,53,54,55,56,57,58,59,60,61,62,63,64,65,66,67,68,69,70,71,72]. (**D**) This illustration demonstrates the efficacy of antibiotic treatment in NVEs for *Abiotrophia defectiva*, granulicatella species, *Viridans streptococci*, *S. gallolyticus*, at a penicillin MIC ≥0.5 μg/mL. The duration of therapy once blood cultures have converted to negative is shown. Abbreviations; MIC, minimal inhibitory concentration, wk, week. Refs. [27,46,47,48,49,50,51,52,53,54,55,56,57,58,59,60,61,62,63,64,65,66,67,68,69,70,71,72]. * 2023 ECC guidelines for IE.

The recommended antibiotic regimens are based nearly exclusively on observational studies and not on randomized clinical trials. (Figure 8A–C) These recommendations are founded upon four underlying standards. The first criterion for evaluating the efficacy of a therapeutic regimen is the ability of the regimen to eradicate the pathogen. The second factor is the administration of a prolonged course of therapy, which may last for several weeks rather than just a few days. The third is that the dosage should be intensive to ensure that the patient receives an adequate amount of the drug over the course of treatment. The fourth is source control. In general, a combination of vancomycin and ceftriaxone represents a reasonable empirical therapeutic option for patients with native-valve infective endocarditis, pending the results of cultures [46,47,48,49,50,51,52,53,54,55,56,57,58,59,60,61,62,63,64,65,66,67,68,69,70,71,72].

The application of beta-lactam antibiotics represents the primary mode of definitive therapy for strains that are sensitive to the treatment. In the absence of contraindications, these drugs are the preferred choice over others unless the patient is unable to tolerate them without adverse effects or has a substantiated prompt (type I) hypersensitivity reaction. In the event that a patient develops IE induced by a penicillin-resistant strain of *Viridans streptococcus*, including *S. gallolyticus*, *Abiotrophia* species, or *Granulicatella* species, a combination of penicillin or ceftriaxone plus gentamicin can be employed as a therapeutic option. While vancomycin monotherapy represents an alternative treatment option, it should be acknowledged that its use in this context is less well-established than that of other drugs [46]. Figure 8A,B is concerned with the antibiotic treatment of NVE sustained by *Viridans streptococci* and *Streptococcus gallolyticus*.

### 5.1. Methicillin-Susceptible Strains of S. aureus

In the case of infective endocarditis caused by methicillin-susceptible strains of *S. aureus* (MSSA), an antistaphylococcal penicillin (e.g., oxacillin) is the medication of preference. Randomized controlled trials have indicated that the combination of an antistaphylococcal penicillin with either gentamicin or rifampin does not result in superior clinical outcomes and is deemed to have an increased risk of adverse events. Consequently, this two-pronged approach is not recommended [19,20,47,48]. In the event that patients with MSSA are unable to receive penicillin, cefazoline represents a reasonable alternative. This is based on the findings of studies that have demonstrated that cefazolin does not cause adverse reactions in these patients. These studies have also demonstrated the efficacy and tolerability of cefazolin in the treatment of MSSA infection [19,49,50,51]. A significant drawback associated with the antibiotic cefazolin is the potential occurrence of an “inoculum effect”. This phenomenon, defined as a rise in the minimum inhibitory concentration (MIC) of the antibiotic in broth culture to 16 μg per milliliter or higher at an inoculum concentration of 5 × 10^7^ CFU per milliliter (100 times the standard inoculum concentration of approximately 5 × 10^5^ CFU per milliliter), necessitates careful consideration [52]. It has been demonstrated that the inoculum effect, which may be at least in part attributed to the hydrolysis of cefazolin by staphylococcal penicillinase, may be associated with clinical failure [53].

In the treatment of NVE caused by methicillin-resistant *Staphylococcus aureus* (MRSA), daptomycin or vancomycin monotherapy is recommended, according to the literature [54,55]. Nevertheless, there is as yet no empirical evidence that the effectiveness of concurrent antibiotic therapy can be demonstrated. A randomized trial was conducted to evaluate the efficacy of vancomycin (or, in eight patients, daptomycin) alone or in combination with an anti-staphylococcal beta-lactam antibiotic (primarily flucloxacillin) for the treatment of methicillin-resistant *Staphylococcus aureus* (MRSA) bacteremia. This study involved a total of 363 patients, including 42 individuals with infective endocarditis. The results demonstrated that concurrent administration of these two medications did not result in any advantage in terms of the primary composite outcome, namely, 90-day mortality, 5-day persistence of bacteremia, microbial relapse, or microbiological treatment failure [56]. In this RCT studying patients receiving concurrent antibiotic therapy, those who died within 90 days had a higher mortality rate despite the rapid clearance of their blood cultures. Furthermore, these patients experienced a significantly higher incidence of acute kidney injury [56]. Based on anecdotal evidence, the combination of a second agent (e.g., ceftaroline) with vancomycin or daptomycin may be beneficial for patients who have persistent bacteremia or who do not respond to the treatment. Nevertheless, the optimal utilization of concurrent antibiotic therapy remains uncertain [57,58,59]. Figure 8C illustrates the use of antibiotic therapy in the treatment of NVE sustained by methicillin-susceptible *Staphylococcus aureus*.

### 5.2. Enterococci 

It is recommended that therapies based on the association of antibiotics be applied in the treatment of enterococcal IE. The standard treatment for decades has been penicillin or ampicillin in combination with a low-dose, synergistic gentamicin. The efficacy of this regimen is constrained by gentamicin toxicity and an increasing prevalence of high-level resistance to gentamicin, which suggests a lack of synergy. Observation data indicates that a six-week treatment regimen of ampicillin and ceftriaxone represents a viable alternative to treat infectious endocarditis caused by ampicillin-susceptible strains of *E. faecalis*, particularly in the absence of contraindications [19,20,42,49,60]. In instances where the ampicillin-gentamicin combination therapeutic approach is utilized, the results of a two-week combined treatment followed by four to six weeks of ampicillin monotherapy have demonstrated comparable outcomes to those achieved by the standard concurrent antibiotic regimen over an equivalent four-to-six-week treatment duration. Additionally, the ampicillin–gentamicin approach exhibited reduced toxicity [42,61,62,63].

It is strongly advised that molecular biology be integrated with microbiology in the context of shared decision-making, with the involvement of microbiological specialists. The use of combined intravenous therapy is generally preferred over monotherapy in order to reduce the likelihood of resistance and to ensure antimicrobial synergism [64]. The laboratory information is encouraging, but the evidence from clinical studies is limited with regard to the use of combination beta-lactam therapy for this indication. Additional investigation is required to ascertain the potential advantages of beta-lactam combination therapy in comparison to monotherapy for the treatment of Gram-positive blood infections. Nevertheless, there is evidence to suggest that combining therapy may be beneficial in cases of bacteremia unresponsive to standard antibiotic treatment [64]. It should be noted that the only exceptions to this rule are *S. aureus* and *E. faecalis*, as they are susceptible to methicillin. There are a range of alternative treatment options for infections that have developed resistance to vancomycin, including linezolid, tigecycline, and daptomycin [65,66].

Despite the low prevalence of beta-lactamase resistance among *Enterococcus faecalis* infections, a recent study employed reverse transcription polymerase chain reaction (RT-PCR) to identify the presence of antibiotic resistance genes (CTX-M, Van A, and Van B) within *E. faecalis* isolates obtained from children with bacteremia. In sharp contrast, the pathogenic *Escherichia coli* ST131 has the ability to actively secrete the CTX-M-15 β-lactamase [67]. A study conducted by Sulainam et al. [67] revealed that 91.67% of *E. faecalis* isolates demonstrated susceptibility to levofloxacin (95% CI, 88.33–94.99), 83.33% to amoxicillin and clavulanic acid, and 66.67% to vancomycin (95% CI, 62.34–70.99). The study also indicated that 53.33% of *E. faecalis* isolates demonstrated intermediate susceptibility to vancomycin. The isolates demonstrated susceptibility to erythromycin (67%), gentamicin (58.33%), ampicillin (50%), and cefotaxime and ceftriaxone (33.33%), respectively. In contrast, only 25% were sensitive to vancomycin.

The findings of the study indicate that 88.89% of the nine vancomycin-resistant isolates were associated with the *Van A* gene, as determined through real-time PCR analysis (*p* < 0.001). It is noteworthy that two crucial aspects merit further examination. First, 77.78% of the isolates displayed production of the *Van B* gene, as identified by real-time PCR (*p* < 0.001). In a subsequent analysis, it was demonstrated that all *E. faecalis* isolates resistant to cefotaxime and ceftriaxone were found to possess the CTX gene, as determined by real-time PCR (*p* < 0.001) [67]. A significant number of antibiotic resistance genes in bacteria have been attributed to genetics in recent investigation. It is widely recognized that the transfer of genetic material between bacteria, either via transformation or transduction, is responsible for the vast majority of instances of antibiotic resistance observed in bacterial strains [68,69,70].

In the context of the rising prevalence of antibiotic resistance, there has been a notable increase in interest within the field of microbiological research in the use of bacterial factors as targets for immunotherapy. The rationale for focusing on bacterial factors is that they play a significant role in an organism’s ability to colonize, infect, and ultimately cause disease (see [71]). MSCRAMMs are currently the subject of significant scrutiny due to their pervasive prevalence and distinctive capacity to facilitate the ictal infection, including endocarditis, in a wide range of pathogens, both traditional and opportunistic [71,72]. Of particular interest is their role in these processes. Unfortunately, complications have arisen in the isolation and definition of MSCRAMMs from *E. faecalis*, which has yielded limited success due to this microorganism’s inability to adhere to ECM proteins under laboratory growth conditions. This contrasts with the behavior exhibited by its relatives, such as staphylococci and streptococci, which demonstrate enhanced aggressiveness. Figure 8D is a detailed illustration of the antibiotic treatment of NVE sustained by *Abiotrophia defectiva, Granulicatella* species, *Viridans streptococci*, and *S. gallolyticus.*

## 6. Clinical Use: Surgical Handling

For patients with NVE, the timing of surgery is critical. Both the likelihood of complications and operative mortality and morbidity often increase when surgery is delayed. It is regrettable that the vast majority of surgeons are only contacted by patients suffering from IE after the failure of medical treatment, when the patients are in refractory cardiac failure or have manifested a major stroke or multi-system organ failure [45,46,73]. In some cases, there is a lack of understanding of the surgical challenges, associated complications, and postoperative clinical outcomes of these cases. The difficulty in identifying the causative pathogen, in conjunction with the aforementioned delays in surgical referral, contributes to a further delay in the patient’s treatment. Patients are frequently offered surgery at a late stage, when their clinical status is significantly compromised, and when the risk of complications during the operation is elevated [45,46,73].

The strategy employed in the treatment of IE is of paramount importance for the survival of the affected individual. The decision regarding the optimal timing for the surgical intervention in question is the result of a shared, multidisciplinary deliberation process. A combination of clinical and echocardiographic assessments enables an accurate determination of the location, extent, and severity of the infectious field, which can include the mitral valve or the aortic valve. It is the responsibility of the multidisciplinary team to address three primary concerns: the presence of an uncontrolled infection, heart failure, and the prevention of embolism [1,4,31,46,73,74,75]. A persistently elevated temperature for a period of five to seven days in the absence of a negative blood culture suggests a state of uncontrolled infection, with the concomitant possibility of local abscess, extensive vegetation, a false aneurysm, fistula formation, and dehiscence of a prosthetic valve. In such instances, it is strongly recommended that emergency surgery be performed. By contrast, in instances where the infection is caused by fungi, multidrug-resistant organisms, or *Pseudomonas aeruginosa*, surgical intervention may be a viable option [1,4,31,40,41,45,46,73,75].

A prospective cohort study of patients with native-valve infective endocarditis revealed that an indication for surgical intervention, irrespective of the success of the procedure, was an independent predictor of mortality [1,4,31,45,46,73,75,76]. The optimal timing of valve surgery remains poorly defined and is a highly individualized decision that is best made by an experienced multidisciplinary team [75,77]. An RCT examined the efficacy of early surgical intervention during the initial hospitalization period, within 48 h of randomization in 37 patients, compared to conventional treatment in 39 patients. The trial evaluated patients diagnosed with endocarditis on the left side of the heart, severe valvular regurgitation (without heart failure) and large vegetations (>10 mm in diameter) [78]. The early surgical intervention demonstrated a notable reduction in the risk of the combined endpoint of in-hospital mortality or embolic events within six weeks following randomization. However, this observed benefit was largely attributed to a decline in the risk of systemic embolism. It is important to note that the trial was limited by the inclusion of a relatively healthy patient population, with few underlying comorbidities. Additionally, the study population was biased towards patients with streptococcal infections and mitral-valve infective endocarditis. The results of two meta-analyses indicate that early surgery, in comparison to conventional therapy (i.e., medical treatment or late surgery after >20 days), is associated with a reduction of mortality from any cause from 40 to 60% [79,80]. However, the optimal method for identifying patients who are most likely to benefit from early valve surgery remains uncertain.

It should be noted that heart failure can result from infection of the mitral or aortic valve, or extensive aortomitral localization of endocarditis with valvular dysfunction. In addition, clinical manifestations and echocardiographic findings may suggest the presence of severe acute regurgitation or obstruction of the valve in the setting of cardiac failure. It is, therefore, important that appropriate medical treatment be administered in a timely manner. In contrast, the presence of pulmonary edema and cardiogenic shock—which are not responsive to medical treatment—may also be observed. Additionally, some patients may present with a fistula into a cardiac chamber or pericardium. In the event that cardiac failure can be managed with medical treatment, elective surgery may be planned. Conversely, urgent surgical intervention is indicated when the degree of cardiac failure is more severe. In the event that the patient exhibits signs of poor hemodynamic tolerance, accompanied by early MV closure or pulmonary hypertension, it is imperative that surgical intervention be promptly initiated. *Enterococcus faecalis* is responsible for the development of IE, which is caused by bacteria that have a specific mode of reproduction, or biogenesis, that allows them to colonize and cause disease. It is noteworthy that a considerable proportion of IE patients require surgical intervention. Consequently, to fully comprehend the severity of disease caused by these bacteria, it is imperative to standardize language and adhere to specific units and metrics. The most commonly encountered strain of *Enterococcus faecalis* is responsible for both native NVE and prosthetic valve endocarditis in elderly patients or those with chronic disease who require a rapid surgical procedure [28,81]. Typical *E. faecalis* lesions are often progressive, forming large abscess cavities involving one or more valves. In the most aggressive forms of IE, extensive portions of the heart, such as the aortic root, intervalvular fibrosa, and cardiac trigones, are destroyed [9,10,40,42].

In cases where surgical intervention is deemed necessary, the only effective method of preventing embolism is emergency surgery. It can be observed that there are a number of factors that increase the risk of embolism. These include the following conditions: manifestations of cardiac failure, persistent infection or abscess, involvement of the MV or aortomitral junction with vegetations larger than 10 mm, or isolated very large vegetation larger than 15 mm. The occurrence of an embolic episode, whether single or multiple, during the initial two weeks of therapeutic intervention is suggestive of an inadequately controlled infection [4,18,31,45].

**Figure 9:**
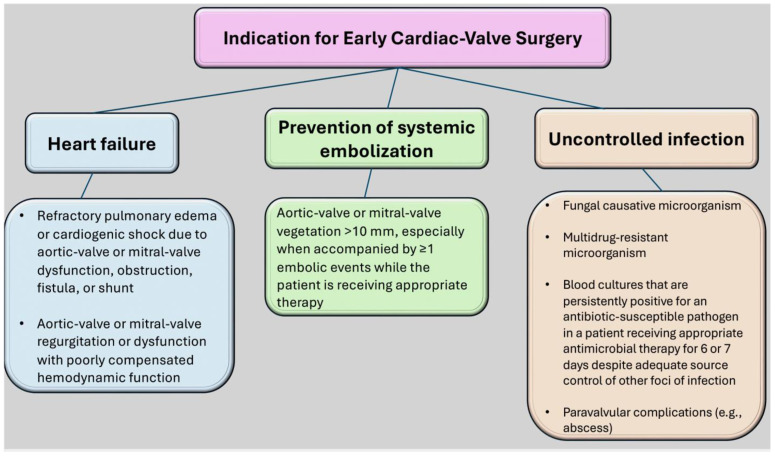
Indication for early surgery. Refs. [4,27,28,31,40,73,77].

It is imperative that the treatment for the infected valve be initiated within 24 h following the completion of diagnostic procedures, given that this is the timeframe in which emergency surgery can be conducted. In the case of patients whose condition is considered urgent, surgery should be performed within a few days of the indication for such treatment. It is recommended that elective surgery be performed at least one to two weeks after the initiation of antibiotic therapy. Elective surgery should be performed after at least one to two weeks of antibiotic therapy. The type of surgical intervention employed is contingent upon the extent of the lesions. When only one leaflet or one scallop is involved, a conservative approach may be considered. However, when there is more extensive involvement of the valve, valve replacement is required [4,18,31,45]. Figure 9 is devoted to the indication to dictate the early cardiac valve surgery.

It is therefore evident that the early involvement of an experienced cardiac surgeon is of the utmost importance in order to determine the optimal surgical option and timing, with the aim of providing the best possible outcome for patients with IE. For instance, the incidence of stroke is markedly elevated during the first two weeks of antibiotic therapy and in patients presenting with left-sided infective endocarditis, particularly those exhibiting valvular lesions within the mitral position. The decision to proceed with either a replacement or repair surgery is guided by the extent of the lesions that define the infectious focus. Furthermore, the potential for mitral valve repair in lieu of replacement can only be fully evaluated following a comprehensive discussion among experienced surgeons and echocardiologists [4,31,45].

**Figure 10:**
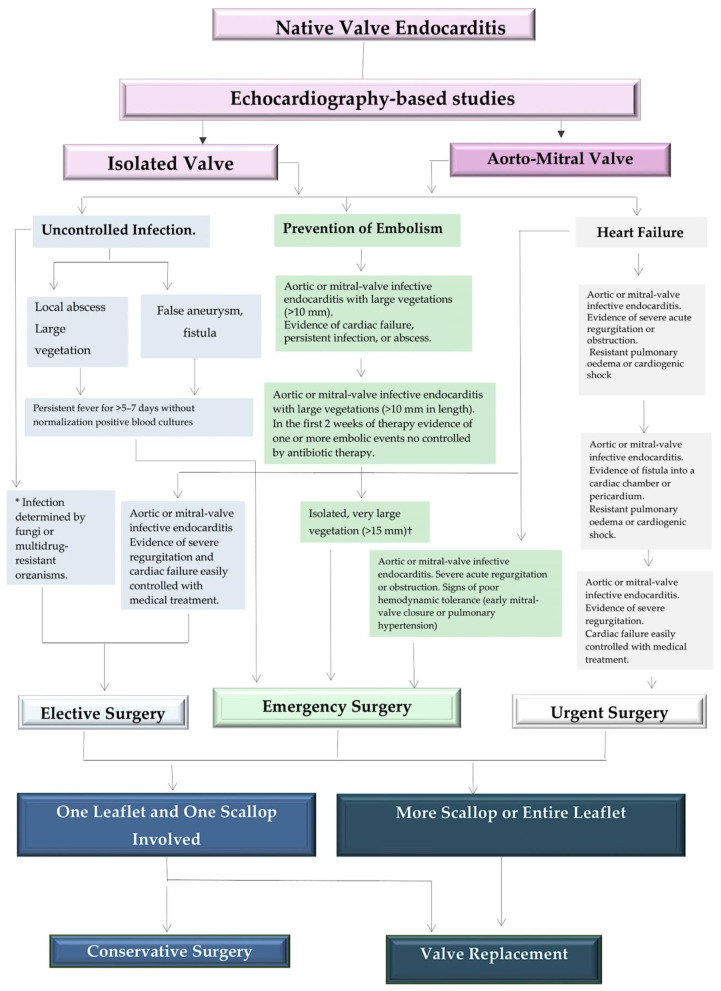
Indications for surgery of isolated or complex valve endocarditis. This illustration presents a summary of the 2023 ESC Guidelines for the management of endocarditis, 2023 Duke-International Society for Cardiovascular Infectious Diseases Criteria for Infective Endocarditis: Updating the Modified Duke Criteria, JCS 2017 guideline on prevention and treatment of infective endocarditis and 2016 The American Association for Thoracic Surgery (AATS) consensus guidelines: surgical treatment of infective endocarditis: executive summary The guidelines are presented in the form of a flowchart, which depicts three different pathways (light blue, light green, and light grey box). according to the degree of urgency. The clinical presentation and imaging findings are described in each pathway. The algorithm used to inform decision-making regarding elective, urgent, or emergency surgery is presented in light blue, light green, or gray boxes, respectively, in the flow diagram. The decision regarding the surgical options (i.e., repair or replacement) is based on the clinical and anatomic findings on preoperative imaging. In the event that the IE is confined to a limited region of the valve leaflets, mitral valve repair should be considered. In cases of extensive anatomic involvement of the valve, surgical mitral valve replacement is the recommended surgical approach. The timing of surgery should be determined through a collaborative, interdisciplinary approach. In the context of emergency surgery, the infected valve must be treated within 24 h of the completion of the diagnostic workup. In the case of patients whose condition is urgent, surgery should be performed within a few days of the indication. Elective surgery should be delayed for a minimum of one to two weeks following the initiation of antibiotic therapy. Refs. [4,19,20,27,28,31,49,77]. * higher frequence of events. † For an indication of the necessity for emergency surgical intervention, please direct your attention to Figure 9.

It has been demonstrated that mitral valve repair can result in improved long-term survival and functional outcome in comparison to valve replacement; as a consequence, a heart team approach has become a crucial element in the success of mitral valve endocarditis treatment. The use of the Society of Thoracic Surgeons (STS) risk scoring system [82,83] can be considered a valid adjunct in discussions with other colleagues, offering a means of objectively defining the operative risk and allowing a more accurate estimation of the intraoperative risk. Infectious disease specialists are likewise essential team members, contributing to the delivery of critical expertise in matters such as the selection of appropriate antibiotics or antifungal agents, as well as their optimal timing and dosage. Furthermore, infectious disease experts can provide invaluable assistance in managing antibiotic-resistant organisms or complications associated with the prolonged use of antibiotics. In addition to the previously mentioned disciplines, the heart team should include experts in internal medicine, nephrology, obstetrics, and geriatrics. It is, therefore, essential that the decision-making derived from a multidisciplinary approach be centered upon the patient’s individual characteristics and that the relative specialist address special circumstances [75,82,83,84]. A substantial proportion of individuals diagnosed with endocarditis are drug abusers; as such, a microbiologist should be consulted alongside a counsellor. A multidisciplinary approach to decision-making is evident in this case; however, it should be noted that the individual characteristics of the patient are of the utmost importance, with each relative specialist addressing the special circumstances that may arise in their respective fields. Notably, drug abusers constitute a significant proportion of the endocarditis population [85]. Consequently, incorporating the advice of a microbiologist, in addition to specific counseling, into the diagnostic process for these cases is advised in order to facilitate an appropriate diagnosis and subsequent treatment. It is imperative to consider the specific needs of young women of childbearing age, particularly in the context of valve replacement. In this context, the use of anticoagulants is contraindicated and a more detailed approach to counseling and discussion regarding the strategy for replacement of the valve is required [86,87,88,89,90,91,92]. In a comparable manner, patients who require long-term dialysis should be evaluated by a nephrologist prior to surgical intervention. Furthermore, an appropriate plan regarding the utilization of hemofiltration during the immediate postoperative phase should be established and discussed [4,45,74]. Figure 10 illustrates the algorithmic approach to treating NVE.

## 7. Discussion

### 7.1. A Cursory Examination of Areas of Incertitude

It is evident that the modified Duke criteria employed for the clinical diagnosis of IE are not contingent on the results of molecular diagnostic testing [14,15,28]. It will be necessary to consider the role of these methods in diagnosis as their accuracy improves and becomes more widely available.

It remains uncertain whether the use of routine brain magnetic resonance imaging (MRI) in conjunction with other advanced imaging techniques such as PET in combination with CT, or PET-CT results in enhanced diagnostic, therapeutic, and outcome outcomes in patients presenting with NVE. It is established that MRI is a more sensitive technique for the detection of central nervous system injuries than computed CT. In vivo identification of asymptomatic embolic injuries patients with suspected infective endocarditis represents a minor supportive criterion for diagnosis in conjunction with clinical criteria and imaging studies [9,30,34,35,42,75,93]. It has been proposed that a routine MRI may serve as a method for detecting silent central nervous system injuries in patients who are eligible for valvular surgery [4,31,45,77,91]. Nevertheless, the impact of this approach on clinical outcomes remains to be determined.

The existing body of data from RCTs does not permit a clear understanding of the benefits and risks associated with the administration of oral antimicrobial agents in the context of infective endocarditis. A trial [94], known as the Partial Oral Treatment of Endocarditis (POET) investigation, revealed that in individuals with infective endocarditis on the left aspect of the heart and whose condition had been stabilized, the administration of oral antibiotics after an inaugural course of intravenous antibiotics was noninferior to conventional intravenous antibiotic therapy at the conclusion of six months of follow-up. Subsequent longer-term follow-up did not reveal any adverse consequences of oral step-down therapy [95]. Nevertheless, a mere 20% of the individuals subjected to screening were ultimately enrolled. Additionally, only a small number had an *S. aureus* infection, with no instances of MRSA. Further information is required in order to elucidate the safety and efficacy of said methodology across a range of clinical setup [96].

The optimal management of timing of surgery for individuals with IE, the circumstances under which surgery should be postponed, and the factors predictive of surgical mortality and poor outcomes require further clarification. The majority of guidelines recommend postponing valve surgery for a minimum of four weeks in patients with substantial embolic central nervous system lesions or intracranial hemorrhage [4,16,19,20,31,45,49,73,74,75]. Nonetheless, it is feasible to proceed with early surgical intervention in carefully selected patients despite these conditions [4,31,45,97]. In patients presenting with small embolic cerebral injuries, those measuring up to a maximum of 2 cm in diameter without evidence of hemorrhage or significant neurological deficits, such an approach is deemed safe.

Cerebral events have been identified as a potential complication in approximately 55% of cases of infective endocarditis and in up to 36% of cases of mitral valve endocarditis.

Of the aforementioned complications, ischemic and hemorrhagic stroke, transient ischemic attack, silent cerebral embolism, mycotic aneurysm, brain abscess, and meningitis are most commonly diagnosed. Individuals suffering with mitral valve endocarditis, particularly those with an *S. aureus* infection, may present with large mobile vegetations of 15 mm in diameter. These vegetations are associated with an elevated risk of embolic events and are a significant independent prognostic factor for mortality following surgery.

Both CT and MRI of the brain are highly accurate techniques for evaluating cerebral abnormalities, including embolic events in mitral valve endocarditis [98,99,100,101,102,103,104,105,106].

It has been demonstrated that patients who have been diagnosed with acute ischemic stroke and who also have a coexisting intracranial aneurysm experience inferior clinical and safety outcome in comparison to patients with neither condition when undergoing intravenous thrombolysis or endovascular thrombectomy, or a combination of the two procedures. In comparison to intravenous thrombolysis, endovascular thrombectomy yields superior functional outcomes, although it is associated with higher rates of postreperfusion intracranial hemorrhage. Furthermore, the advent of advanced histological clot analysis following endovascular thrombectomy offers a novel approach to the identification of the underlying cause of cryptogenic strokes, which can be classified according to the results of a meticulous morphological and histological examination of endovascular thrombectomy-retrieved clots. This examination may include Gram staining, which can assist in the classification of the clot. An appreciation of the composition of the clot may prove clinically beneficial in the early diagnosis of IE and the subsequent development of treatment plans [107,108].

A multitude of scoring systems have been devised with the aim of anticipating the risk of mortality or postoperative complications in patients with infective endocarditis. However, despite their widespread use, several limitations have been identified. These include a lack of robust sample sizes, a reliance on retrospective data, and the necessity to consider changes in surgical practice over time, which may extend over several decades, and a lack of large-scale external validation. Consequently, it has proven challenging to evaluate the accuracy of these systems in a rigorous and systematic manner [109,110,111,112,113].

### 7.2. How Should We Interpret the Guidelines?

**Figure 11:**
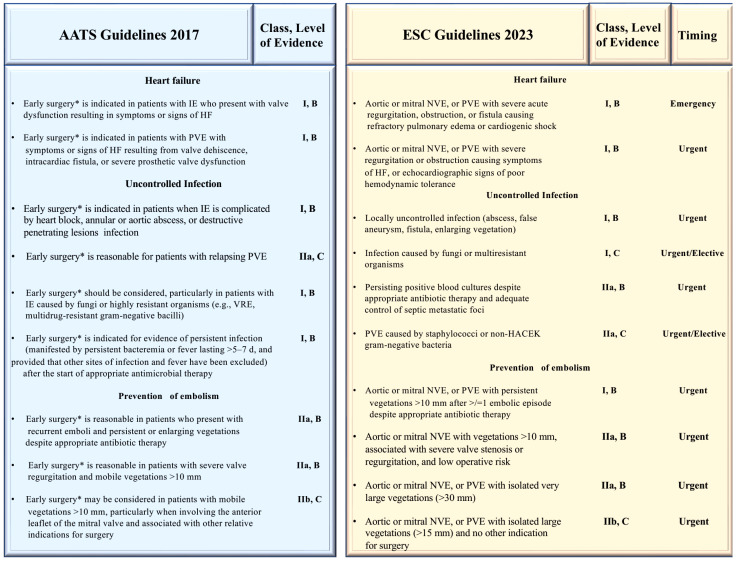
Guidelines for Infective Endocarditis. Refs. [28,77]. * For an indication of the necessity for early surgical intervention, please direct your attention to Figure 9.

The American Heart Association, the European Society of Cardiology, the Japanese Society of Cardiology, and the American Association for Thoracic Surgery have each published guidelines for the diagnosis and management of IE [19,20,27,28,49,75,77,99]. The aforementioned guidelines are, for the most part, concordant in their recommendations, though there are a few differences of note, particularly in regard to antimicrobial therapy, imaging modalities, and indications for and timing of surgical procedures. The recommendations set forth herein are generally consistent with the aforementioned guidance. Figure 11.

## 8. Conclusions

From the perspective of purely clinical indicators, the coexistence of bacteremia and a murmur in a febrile patient strongly points to the potential presence of NV. Upon initial assessment, patients must undergo investigation to ascertain the fulfilment of at least three of the established Duke criteria, which include fever, the isolation of two distinct bacterial cultures indicative of the causative pathogen, and, in the case of elderly individuals, the presence of a primary focus of pyelonephritis, which often occurs in conjunction with IE caused by *Enterococcus faecalis*. Nevertheless, it is possible that the patients did not meet the criteria for a major condition. Furthermore, the presence of aortic stenosis, a preexisting cardiac condition, further supports the possibility of endocarditis.

To meet the criteria for a diagnosis of infective endocarditis—persistent positive blood culture(s)—it would be prudent to obtain an additional blood culture(s). It is imperative that an echocardiogram be conducted as soon as possible to accurately diagnose the nature of the valvular lesion, determine the presence of vegetations, and ascertain the extent of any complications associated with infective endocarditis. Despite the greater sensitivity of TEE for identifying valvular vegetation and paravalvular complications, it is prudent to commence with TTE, as its noninvasive nature, ease of implementation, and superior myocardial function information (e.g., ejection fraction) render it a superior choice. In the event that a TTE is found to be either negative or inconclusive, a TEE should be conducted, given the strong suspicion that the patient may be suffering from infective endocarditis. In the event that TEE is non-diagnostic and the suspicion for infective endocarditis remains high, it would be advisable to repeat the examination several days later.

A multidisciplinary team would be constituted for the purpose of providing care, with specialists in cardiology, cardiovascular surgery, and infectious disease included among its members. The prompt administration of combination antimicrobial therapy is essential in the treatment of presumptive NVE. In the presence of *E. faecalis* bacteremia, although the susceptibility of the isolate to gentamicin should be confirmed, the patient’s age, diabetes, and chronic kidney disease place the patient at high risk for acute kidney injury from gentamicin. It is therefore recommended that the initial treatment should be based on the administration of ampicillin and ceftriaxone, in accordance with the relevant clinical protocols. Obtaining blood cultures is essential to confirm the clearance of bacteremia with therapy. Furthermore, a meticulous evaluation of the patient is necessary to identify any indications for immediate valve surgery, including those related to neurological dysfunction and complications [114,115,116,117,118,119,120]. It is recommended that antimicrobial therapy be continued for a period of six weeks following the conversion of blood cultures to a negative result. It is similarly vital to consider colonoscopy as a potential avenue in the diagnostic pathway. Some evidence suggests that, in a manner analogous to NVE in the elderly population, enterococcal infective endocarditis may be causally related to colonic neoplasms with spinal infection as first manifestation [9,42,113,120,121,122,123,124,125,126,127].

Nevertheless, further research is required to substantiate the aforementioned association between the use of a novel, development, and validation model, based on a nomogram, and in-hospital mortality in ICU patients with infective endocarditis [112,128,129]. The selection of essential variables for the development of a machine learning-based model for the prediction of mortality in IE may be effectively established as demonstrated by the CatBoost model, which demonstrated the optimal predictive performance [130].

**Figure 12:**
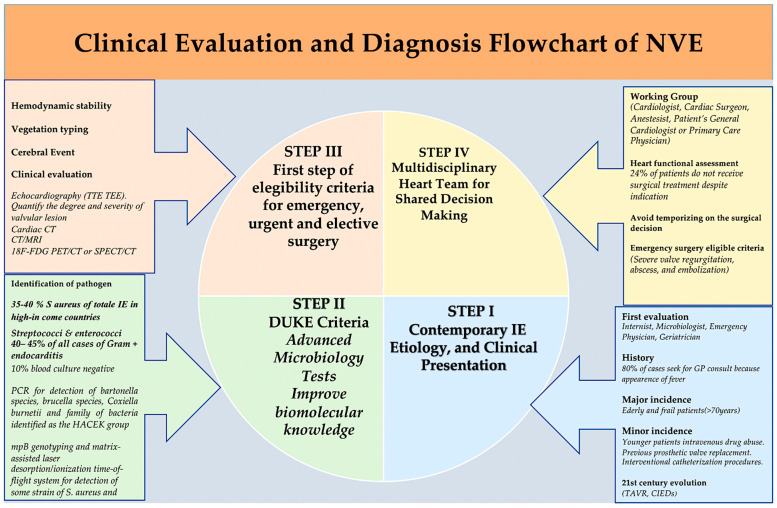
Clinical evaluation and diagnosis flowchart of native valve endocarditis.

In conclusion, this review highlights the persistent high mortality rates of NVE and the need for early diagnosis and aggressive treatment. Clinicians should be vigilant for symptoms such as bacteremia and heart murmurs in febrile patients. Future research should focus on developing more effective treatment protocols and preventive measures. The following Figure 12 provides a summary of the clinical evaluation and diagnosis flow chart in patients presenting with NVE.

## Data Availability

Not applicable.
